# Energy deprivation-induced AMPK activation inhibits milk synthesis by targeting PrlR and PGC-1α

**DOI:** 10.1186/s12964-022-00830-6

**Published:** 2022-03-05

**Authors:** Zhihui Wu, Qihui Li, Siwang Yang, Tenghui Zheng, Jiayuan Shao, Wutai Guan, Fang Chen, Shihai Zhang

**Affiliations:** 1 Guangdong Province Key Laboratory of Animal Nutrition Control, College of Animal Science, South China Agricultural University, Guangzhou, 510642 China; 2 College of Animal Science and National Engineering Research Center for Breeding Swine Industry, South China Agricultural University, Guangzhou, 510642 China; 3 Guangdong Laboratory for Lingnan Modern Agriculture, South China Agricultural University, Guangzhou, China

**Keywords:** AMPK, Mammary epithelial cells, Milk protein, Milk fat, PrlR, PGC-1α

## Abstract

**Background:**

The mammary gland is responsible for milk production and secretion, which is critical for neonatal health during lactation. Lactation efficiency is largely affected by energy status with unclear mechanism.

**Results:**

In the current study, we found that synthesis of milk fat and protein was significantly inhibited under energy-deficient conditions, which is accompanied with AMP-activated protein kinase (AMPK) activation. Modulating the AMPK signaling pathway directly or indirectly affects the synthesis of milk fat and protein. Besides mammalian target of rapamycin complex 1 (mTORC1) signaling in the regulation of milk synthesis, we discovered that AMPK mainly regulates the synthesis of milk protein through prolactin signaling. Mechanistically, AMPK triggers the ubiquitination of prolactin receptor (PrlR) through regulating the activity of β-transducin repeat-containing protein (β-TrCP, an E3 ligase). Subsequently, PrlR is degraded by the endocytosis process of lysosomes, which further attenuates prolactin signaling. In addition, our results revealed that AMPK activation inhibits milk fat synthesis through decreasing and accelerating de novo synthesis and β-oxidation of fatty acids, respectively. To be precise, AMPK activation inhibits rate limiting enzymes and transcriptional regulatory factors involved in de novo fatty acid synthesis and decreases the acetylation process of peroxisome proliferator-activated receptor gamma coactivator-1 alpha (PGC-1α) to strengthen the oxidation of fatty acids.

**Conclusions:**

Taken together, AMPK regulates the synthesis of milk not only depends on canonical mTORC1 signaling and key rate-limiting enzymes, but also through manipulating the degradation of PrlR and the acetylation of PGC-1α.

**Video Abstract**

**Supplementary Information:**

The online version contains supplementary material available at 10.1186/s12964-022-00830-6.

## Background

The mammary gland is the hallmark of mammals, which is involved in the process of milk synthesis. The nutritional property of maternal milk determines the neonatal growth and health outcomes during breastfeeding. Maternal nutrients not only supply substrates but also provide large amounts of energy for the guarantee of milk synthesis [1]. During early lactation, maternal nutrition intake is usually insufficient to meet the requirement for milk secretion, which leads to a state called ‘negative energy balance’. As a crucial sensor for cellular energy level, AMP-activated protein kinase (AMPK) is activated during the decrease of cellular ATP and the increase of ADP and AMP under energy stress. In addition, AMPK is also activated by the absence of fructose-1,6-bisphosphate (FBP), which is a metabolite of glucose [2]. Preliminary evidence indicated that once activated, AMPK attenuates milk synthesis through the inhibition of enzymes that directly participate in the synthesis of milk fat [3] and milk protein [4, 5]. However, whether AMPK regulates milk synthesis through other critical enzymes or pathways remains largely unknown.

Mammary epithelial cells control milk fat level by balancing fatty acids synthesis and their degradation through β-oxidation system. Current studies mainly focus on the processes of de novo fatty acid (FA) synthesis, which includes FA uptake, activation, intracellular transport, elongation and desaturation, as well as triacylglycerol (TAG) synthesis and lipid droplet formation [6]. A number of rate-limiting enzymes of de novo fatty acid synthesis have been identified as the downstream targets of AMPK, such as acetyl-coA carboxylase alpha (ACACA) (phosphorylation site is S79) and sterol regulatory element-binding protein 1c (SREBP1c) (phosphorylation site is Ser372). ACACA catalyzes the conversion of acetyl-CoA to malonyl-CoA and initiates de novo FA synthesis [7]. SREBP1c is a transcription factor for lipid homeostasis, which regulates milk fat synthesis in cows [8], goats [9] and swine [10]. Currently, whether AMPK regulates milk fat synthesis through β-oxidation system remains less well understood.

Milk protein synthesis is mainly regulated by the process of gene transcription and protein translation. Mammalian target of rapamycin (mTOR) is a master regulator that promotes anabolic process. Previously, mTOR complex 1 (mTORC1) has been identified to control gene transcription and protein translation through regulating its two downstream molecules including ribosomal protein S6 kinase 1 (S6K1) and 4E binding protein 1 (4EBP1). In the mammary epithelial cells of rats [11] and cows [4], inhibition of mTORC1 activity could down-regulate the mRNA expression of genes encoding milk proteins such as alphaS1-casein (CSN1S1) and beta-casein (CSN2) and the process of milk protein translation [5]. Currently, how AMPK regulates milk protein through mTORC1-independent pathway (such as JAK2-STAT5) remains largely unknown.

In our current study, we confirmed that AMPK regulates the synthesis of milk partially through the activation of mTORC1 signaling and expression of rate limiting enzymes. Importantly, we identified prolactin receptor (PrlR) and peroxisome proliferator-activated receptor-C coactivator-1 alpha (PGC-1α) as novel potential targets of AMPK to regulate milk protein and fat synthesis, respectively. We further found that AMPK induces the activity of β-TrCP, which leads to the ubiquitination of PrlR and disruption of the prolactin signaling. AMPK promotes the β-oxidation of milk fatty acids through regulating the acetylation of PGC-1α. Taken together, we identified a novel molecular mechanism that AMPK regulates the synthesis of milk not only depends on canonical mTORC1 signaling and key rate-limiting enzymes, but also through manipulating the degradation of PrlR and the acetylation of PGC-1α.

## Materials and methods

### Cell cultures

HC11 (mouse mammary epithelial cells) used in this study was first cultured in DMEM/F12 medium (Thermo) containing 10% FBS (fetal bovine serum), 0.4% HS (hydrocortisone), 0.4% EGF (epidermal growth factor), 0.4% ITS (Insulin-Transferrin-Selenium) and 1% PS (penicillin–streptomycin) in a cell incubator at 37 ℃ and 5% CO_2_ concentration. When the cells reached to around 80% confluence, they were seeded in culture plates and incubated with the same medium and conditions. When the cells in culture plates reach to approximately 80% confluence again, medium were changed to DMEM/F12 glucose-free medium (Thermo). This step was mainly to deplete the glucose in the cells for subsequent experiments. After 4 h-starvation treatment, cells were assigned into treatment mediums and harvested after 12 h and 24 h for real-time PCR and western-blotting, respectively. The reagents used in this experiment (mainly inhibitors or activators of pathways) are listed in Table 1.

**Table 1:** Reagents for this experiment

Reagent	Company	Cat No
AICAR	Sigma	A9978
Phenformin hydrochloride	Sigma	P7045
Compound C	Sigma	171261
Rapamycin	Sigma	V900930
Pitstop	Sigma	SML1169
EIPA	Sigma	3378
MG132	Sigma	M8699
MA	Sigma	M9281
SB203580	Sigma	HY-10256

### RNA-Seq analysis

Total RNA was extracted using Trizol reagent kit (Invitrogen, Carlsbad, CA, USA) according to the manufacturer’s protocol. The DNA libraries were sequenced on the Illumina sequencing platform by Genedenovo Biotechnology Co., Ltd (Guangzhou, China).

### Lipidomics

After the cells were processed, they were scraped and collected with PBS. The subsequent operations and data processing were completed under the technical guidance and assistance of Novogene Co., Ltd. (Beijing, China).

### siRNA transfection

The siRNA and other reagents used in this experiment were provided by RiboBio (China), and our operation was in accordance with the instructions provided by them. We first selected the one with the highest transfection efficiency from the 3 siRNA products provided by the company, and then used it for subsequent formal experiments.

### Oil red O staining of lipid droplets

To observe the production of milk fat more intuitively, oil red O staining solution (Sangon Bio, Shanghai) was used to observe the lipid droplets. In this experiment, the oil red dye solution used was diluted with double distilled water in a ratio of 6:4, and then filtered through 45-nm filters for 3 to 5 times before it can be used as a working solution. The cells were seeded into 24-well plates and cultured according to the methods mentioned above. They were harvested 24 h after being treated with the treatment medium for oil red staining. In brief, the cells were rinsed 3 times with PBS and fixed with 4% paraformaldehyde for 30 min at room temperature. Then, fresh working solution was added to cells (200 μL per well) followed by rinse with PBS twice. After 6 h of incubation at room temperature, cells were rinsed with PBS until there were no visible pellets before being observed and photographed under a microscope.

### Measurement of triacylglyceride concentration

For quantitative estimation of the synthesis of intracellular and extracellular triacylglyceride, Triglyceride Assay Kit (Nanjing jiancheng, China) was used to detect the concentration of triacylglyceride in this research. The cells were seeded into 12-well plates and cultured as described previously and collected 24 h after being treated with the treatment medium for triacylglyceride determination. Briefly, 100 μL of medium from each well was collected uniformly for the subsequent determination of extracellular triacylglyceride. After rinsing with PBS, 50 μL of RIPA lysis buffer containing 1% PMSF and 1% phosphatase inhibitor was added to each well. After lysis on ice for around 15 min, the mixture in the well was collected as the samples for the subsequent determination of intracellular triacylglyceride. The determination of triacylglyceride concentration was achieved by detecting the OD value of the sample at a wavelength of 510 nm as the instructions suggested.

### Western-blotting

As with the previous steps for determining the triglyceride concentration, the HC11 cells were first fully lysed by RIPA (160 μL per well for 6-well plates). After the cell lysate were denatured by high temperature by using a PCR instrument, they were mixed with the 40 μL loading buffer per well and used as the sample for subsequent experiments. Under the action of electrophoresis, the proteins in the sample were separated by 10% or 12% SDS-PAGE gels. Then, the protein molecules were transferred onto the PVDF membrane in the tris–glycine system. Immediately afterwards, the PVDF membranes were rinsed with TBST 3 times and blocked with 6% skimmed milk for two hours at room temperature. The PVDF membranes were then rinsed 3 times with TBST and incubated under gentle shaking overnight at 4℃ with the primary antibodies. After overnight incubation, the PVDF membranes were rinsed 3 times with TBST and incubated under gentle shaking at room temperature for 1 h with the secondary antibodies. The chemiluminescent signal was detected using super ECL reagents (P1020), and signals obtained were quantified by ImageJ Software (ImageJ 1.52a). All antibodies used in this experiment (including subsequent immunofluorescence and immunoprecipitation experiments) are shown in Table 2.

**Table 2:** Antibodies for this experiment

Antibody	Company	Cat No	Dilution
FASN	Abcam	ab99539	1:1000
ACACA	Abcam	ab72046	1:1000
FABP3	Abcam	ab231568	1:1000
DGAT1	Abcam	ab181180	1:1000
SREBP1	Abcam	ab3259	1:1000
alpha-S1-casein	Santa Curz	sc365929	1:100
beta-casein	Santa Curz	sc166520	1:100
WAP	Santa Curz	sc398276	1:100
Phospho-AMPK	CST	2535	1:1000
AMPK	CST	2532	1:1000
Phospho-mTOR	CST	5536	1:1000
mTOR	CST	2983	1:1000
Phospho-S6K1	CST	9234	1:1000
S6K1	CST	9202	1:1000
Phospho-4EBP1	CST	9451	1:1000
4EBP1	Abcam	ab32024	1:1000
Phospho-JAK2	CST	3776	1:1000
JAK2	CST	3230	1:1000
Phospho-STAT5	CST	4322	1:1000
STAT5	CST	94205	1:1000
Phospho-p38	CST	4511	1:1000
p38	CST	8690	1:1000
SirT1	CST	8469	1:1000
β-TrcP	CST	4394	1:1000
PrlR	Abcam	ab170935	1:1000
PGC-1α	Santa Curz	sc518025	1:100
Acetylated-lysine	CST	9441	1:1000
Anti-ubiquitin	Abcam	ab140601	1:2000
IgG	Santa Curz	sc2025	1:100
β-actin	Abcam	ab8226	1:2000
Goat anti-mouse IgG	ZENBIO	511103	1:5000
Goat anti-rabbit IgG	ZENBIO	511203	1:5000
Goat anti-mouse IgG	Jackson	115545003	1:500

### Immunofluorescence staining

As shown in previous studies, immunofluorescence staining was performed to determine the location and yield of the target protein. We use 96-well plates to culture and process cells for immunofluorescence. The cells were washed, fixed with 4% paraformaldehyde, treated with tiriton X-100, and blocked with 5% BSA, and then incubated with the primary antibodies and the secondary antibodies in turn. After being stained by DAPI, the cells were observed and imaged under a fluorescence microscope.

### Immunoprecipitation

In order to detect the degree of acetylation or ubiquitination of a single protein, we introduced immunoprecipitation to separate the target protein from other proteins, and then used the corresponding antibodies to bind to the target protein. The main steps are as follows: (1) fully lyse the cells cultured in the culture dishes with CHAPS lysis buffer (TIANDZ, China), and centrifuge the lysate to collect the supernatant; (2) take a small part of the supernatant and save it for later use, and add IgG and the target protein antibody to the rest and incubate at 4 °C for 2 h; (3) add the A/G beads (washed and blocked in advance) (Santa cruz, sc2003) to the solution in the previous step, and incubate it overnight at 4 °C; (4) discard the supernatant and add chaps buffer, then bath at 100 °C for 5 min; after centrifugation, the supernatant and loading buffer are mixed. The subsequent operations are basically the same as western-blotting.

### Real-time PCR

The cells were harvested after 12 h incubation for real-time PCR. Total RNA was isolated from cells by using EZ-press RNA Purification Kit (EZ-Bio, Shanghai) according to the protocol. After being tested the purity and integrity via agarose gel electrophoresis, the RNA was then reverse transcribed into cDNA by Color Reverse Transcription Kit (EZ-Bio, Shanghai) including gDNA removers and colored mix. The diluted cDNA was thoroughly mixed with Color SYBR Green qPCR Mix (EZ-Bio, Shanghai) and the primers of the target gene as well as double distilled water to form a 20 μL system for qPCR. The thermal cycling conditions of qPCR reactions were as follows: 95 °C for 1 min followed by 40 cycles of denaturation at 95 °C for 15 s, annealing at 59 °C for 15 s, and extension at 72 °C for 40 s. Primer suquences we used for real-time PCR were listed in Table 3.

**Table 3:** Primer sequences for real-time PCR

Genes	Forward primers	Reverse primers	Size, bp
FASN	AGCACTGCCTTCGGTTCAGTC	AAGAGCTGTGGAGGCCACTTG	94
ACACA	GAAGTCAGAGCCACGGCACA	GGCAATCTCAGTTCAAGCCAGTC	119
FABP3	ACCTGGAAGCTAGTGGACAG	TGATGGTAGTAGGCTTGGTCAT	106
DGAT1	CAGCTGTGGCCTTACTGGTTGA	CGGCACCACAGGTTGACATC	118
SREBP1	AGAAGCTCAAGCAGGAGAACCTGA	ACTTCGGGTTTCATGCCCTCCATA	127
AMPK	AACCTGAGAACGTCCTGCTTGATG	TGACTTCTGGTGCGGCATAATTGG	132
CSNS1	AATTCTTCCAGCTTGGTGCCTCTC	CAATGCCTTCAGGGGTGTCGTAG	105
CSN2	AGGAACAGCAGCAAACAGAGGATG	GGCTGAAGAGGCGGCAGAAAG	136
WAP	ACAGAGTGTATCATCTGCCAAA	ACCAATGTTGACAGGAGTTTTG	102
PCG-1α	CAGAGAGTATGAGAAGCGAGAG	AGCATCACAGGTATAACGGTAG	232
PrlR	CCTGAAATCCACAAATGTCGTT	CATATGGAAGTGTACTGCTTGC	197
PIP	CATCAACAGCACAGGAAAATCA	AGGATGATCATTACAGAGGCAG	158
BCAT	TTTGCTGGAGAAAGAGGATCAG	GCTTCTCTTTTAAGACAGTGGC	91
GPT	CTTCAAGCAGTTTCAAGCAGAG	TTGAGGGAAGGAATACATAGCG	139
GOT	ACAAGAACACACCAATCTACGT	ATAGGGCCGAATGTCCTTAAAA	92
ASCT2	TCCTTTCTAGATCTCGTGAGGA	CTGGACCATGGTTGAATTGATC	150
ASCT1	GCAGGACAGATTTTCACCATTC	CAAGATGATGGCAATTGTGAGG	99
xCT	CTATTTTACCACCATCAGTGCG	ATCGGGACTGCTAATGAGAATT	102
CAT2	CATCGGAATAGTGACGTCCTTA	AAGGCATCATAAGCGTTAAAGC	80
CAT1	CTGTGTTTTGGTCTTACGGTAC	AAAAAGCCTGTCTGTGATTCAC	132
4F2hc	AGGGACTCCTGTTTTTAGCTAC	GTGAAAGATGCTGGACTCATTC	109
LAT2	TGGATCCTTACAAGAACCTTCC	CATTGCAGTGACGTATGCAATA	98
ACTIN	CCACCATGTACCCAGGCATT	CGGACTCATCGTACTCCTGC	190

### Statistical analysis

Data of western-blotting, real-time PCR and TAG concentration were analyzed by one-way analysis of variance using IBM SPSS Statistics 20.0. The graphs were performed and analyzed with GraphPad Prism software 8.0. All data were demonstrated as (mean + SEM, n). *P* < 0.05 was considered as statistically significant and *P* < 0.01 was considered as highly statistically significant.

## Results

### Glucose starvation inhibits milk fat and protein synthesis

**Fig. 1:**
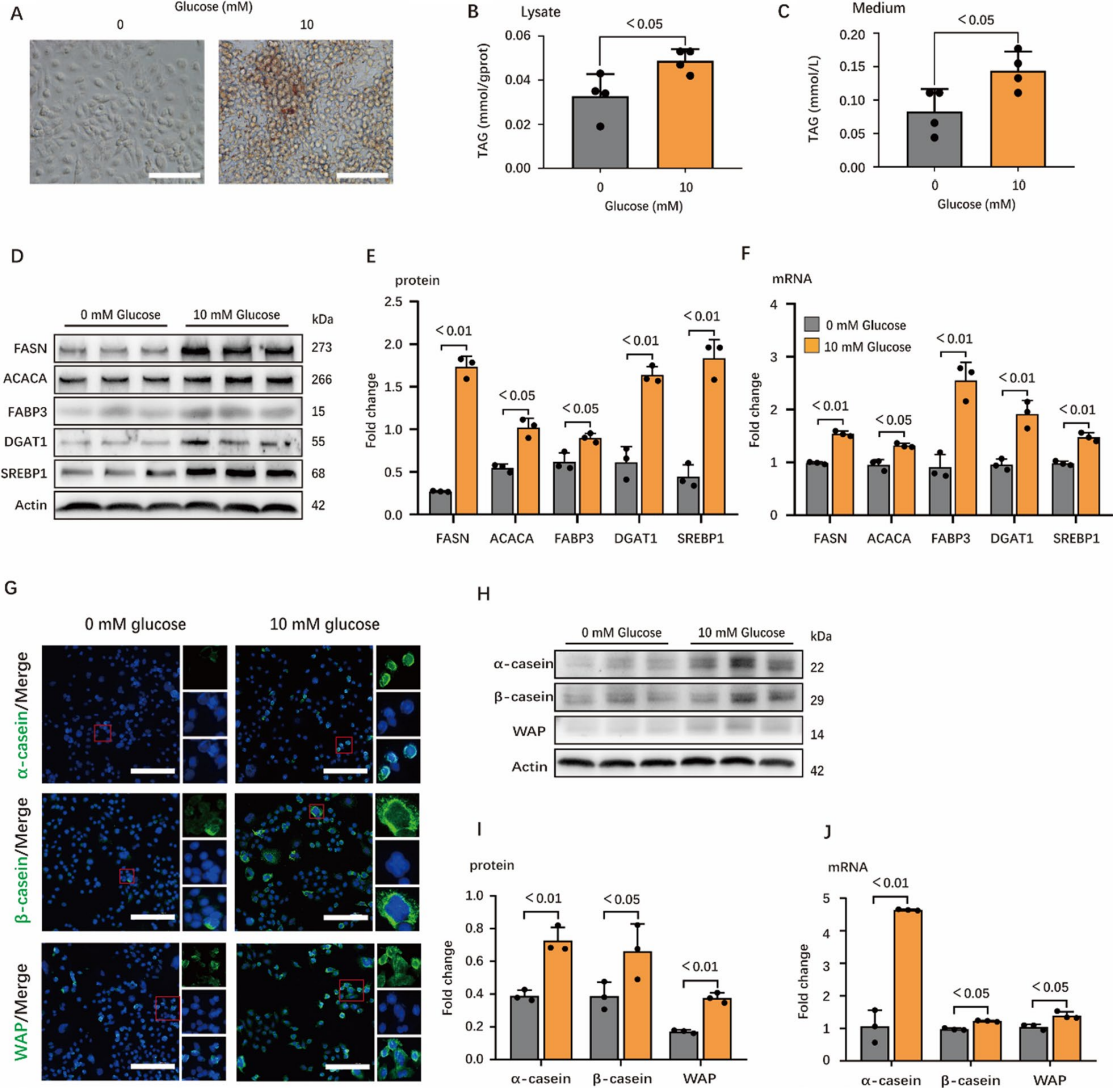
Energy stress suppresses synthesis of milk fat and protein in HC11. **A** Oil red O staining images of HC11 under 0 mM glucose (left) and 10 mM glucose (right). Scale bars are 100 μm. **B** and **C** TAG concentration in cell (**B**) and medium (**C**), n = 4. **D**–**F** Western blots of HC11 lysates. In (**D**), bands were incubated with target proteins including FASN, ACACA, FABP3, DGAT1, SREBP1 and Actin antibodies, n = 3. Protein expressions of target proteins are shown in (**E**), n = 3. **F** Relative mRNA expression of target proteins, n = 3. **G** Confocal microscopy of HC11 immunostained for α-casein (green), β-casein (green), WAP (green) and DAPI (blue). Scale bars, 100 μm. The small red boxes in the figure are typical areas selected, and the three small images on the right are the enlarged images of the target protein, nucleus and merge images in selected areas, respectively. **H**–**J** Western blots and real time PCR of HC11 lysates of genes critical for protein synthesis. In (**H**), bands were incubated with target proteins including α-casein, β-casein, WAP and Actin antibodies, n = 3. Protein expressions of target proteins are shown in (**I**), n = 3. **J** Relative mRNA expressions of target proteins, n = 3. In this figure, cells for oil red O staining, TAG determination, immunofluorescence staining and WB were collected after 24 h incubation with 0 mM and 10 mM glucose mediums and cells for real-time PCR are 12 h. All data with error bars are averages ± SEM. In histograms, the numbers above the column are the p-values marked as a range if significantly different (*P* < 0.05) and each small black dot represents a test value

As a major energy source for epithelial cells of the mammary gland, glucose starvation increases cellular AMP/ATP ratio and triggers energy stress. To understand the effects of energy stress on milk synthesis, we first studied the effects of glucose starvation on milk protein and fat synthesis in mouse mammary epithelial cell line HC11. As expected, glucose starvation dramatically inhibited the milk fat synthesis (Fig. 1A–C). Correspondingly, protein (Fig. 1D, E) and gene (Fig. 1F) levels of critical enzymes (FASN, ACACA, FABP3 and DGAT1) participating in milk fat de novo synthesis and the regulatory factor (SREBP1) were also down-regulated under glucose starvation. We further detected the effects of glucose starvation on milk protein synthesis. Similarly, we observed positive reactions to α-casein, β-casein and WAP in the cytoplasm of HC11 cells treated with 0 mM glucose, while they were greatly inhibited compared with cells treated with 10 mM glucose (Fig. 1G). Milk protein synthesis was significantly inhibited during glucose deficiency, as is shown by the results from proteins (Fig. 1H, I) and genes expression (Fig. 1J) of milk protein (α-casein, β-casein and WAP). In order to confirm the effects of glucose on epithelial cells of the mammary gland, glucose dose-dependent experiment was conducted on porcine mammary gland epithelial cells (pMECs). Similar to the results from HC11 cell line, treating pMECs with different concentration of glucose after glucose starvation dose-dependently increased the milk fat synthesis (Additional file 1: Fig. S1A) with the upregulation of fat de novo synthesis enzymes (Additional file 1: Fig. S1B–C). Collectively, energy stress may inhibit milk fat (also verified in pMECs) and protein synthesis in HC11.

### AMPK activation mimics the negative effects of glucose starvation on milk fat and protein synthesis

**Fig. 2:**
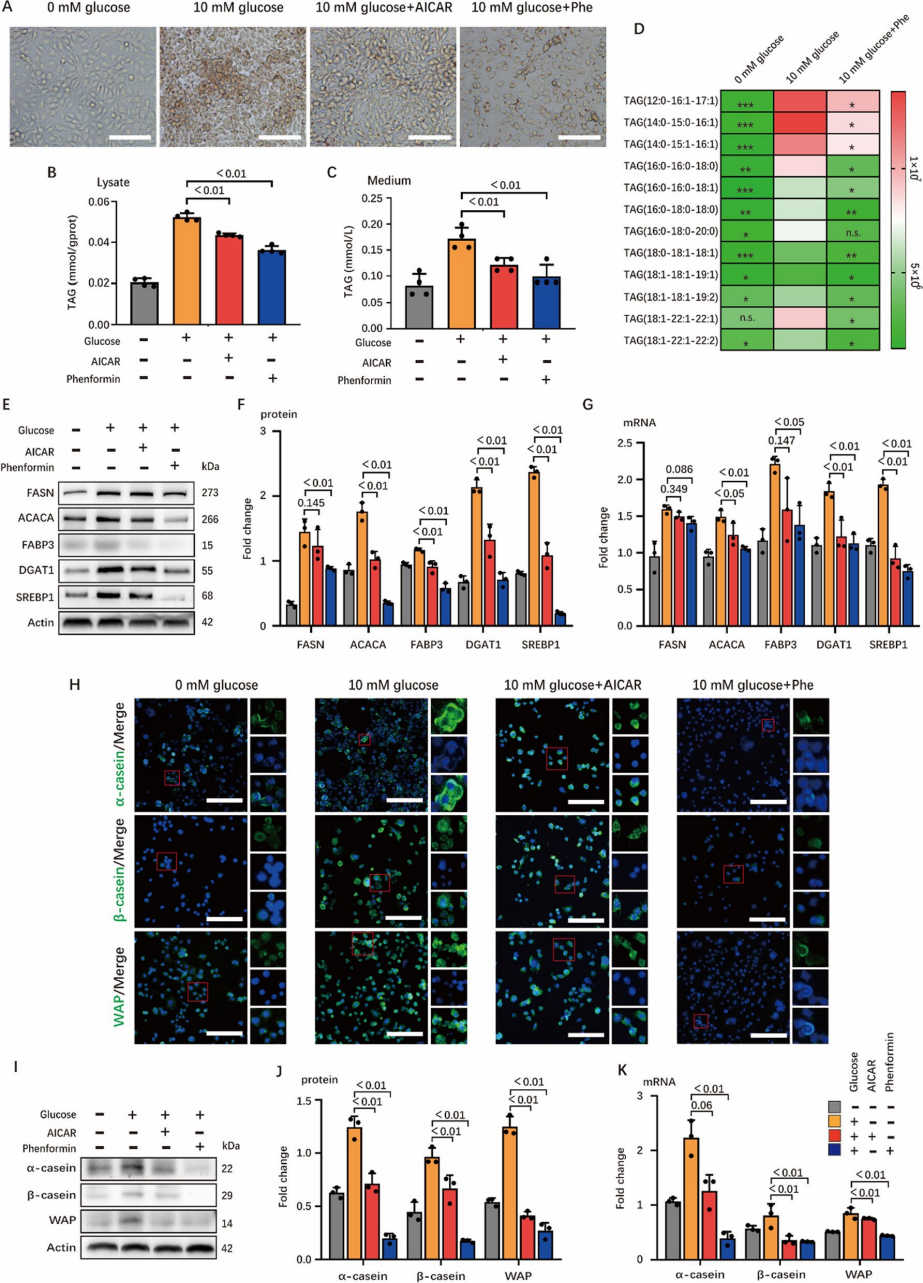
AMPK activation attenuates synthesis of milk fat and protein in HC11. **A** Oil red O staining images of HC11. Scale bars are 100 μm. **B** and **C** TAG concentration in cells (**B**) and medium (**C**), n = 4. **D** Heatmap analysis of up-regulated (red) or down-regulated genes (green) in HC11, n = 3. The 0 mM glucose group and the Phe group were compared with the 10 mM group respectively, * means *P* < 0.05, ** means *P* < 0.01 and *** means *P* < 0.001. **E**–**G** Western blots and real time PCR of HC11 lysates for critical fat synthesis genes. **E** Representative western-blotting bands hybridized with target proteins including FASN, ACACA, FABP3, DGAT1, SREBP1 and Actin antibodies, n = 3. (**F)** is the result of analyzing the WB bands and (**G)** represents data from real-time PCR, n = 3. **H** Confocal microscopy of HC11 immunostained for α-casein (green), β-casein (green), WAP (green) and DAPI (blue). Scale bars, 100 μm. The small red boxes in the figure are typical areas selected, and the three small images on the right are the enlarged images of the target protein, nucleus and merge images in selected areas, respectively. **I**–**K** Western blots and real time PCR of HC11 lysates for critical protein synthesis genes. **I** Representative western-blotting bands hybridized with target proteins including α-casein, β-casein, WAP and Actin antibodies, n = 3. **J** Result of analyzing the WB bands and (**K)** represents data from real-time PCR, n = 3. In this figure, cells for oil red O staining, TAG determination, lipidomics, immunofluorescence staining and WB were collected after 24 h incubation with 0 mM glucose medium, 10 mM glucose medium, (10 mM glucose + 1.5 mM AICAR) medium and (10 mM glucose + 300 μM Phe) medium, and cells for real-time PCR are 12 h. All data with error bars are averages ± SEM. In histograms, the numbers above the column are the *p*-values marked as a range if significantly different (*P* < 0.05) and each small black dot represents a test value

AMPK is a crucial sensor to detect cellular energy status. Glucose starvation was shown to decrease the cellular ATP/ADP ratio (Additional file 1: Fig. S1D) and activated AMPK in HC11 cells (Additional file 1: Fig. S1E–F). To identify the potential role of AMPK on milk fat and protein synthesis, we used compounds 5-aminoimidazole-4-carboxamide ribonucleotide (AICAR; an AMP analogue) and phenformin to activate AMPK to mimic the effects of glucose starvation. Strikingly, treatment with these compounds both highly inhibited (especially phenformin) milk fat synthesis (Fig. 2A–C). Furthermore, the type of triglyceride (LCFA saturation) that decreased during AMPK activation was detected (Fig. 2D). Significant decrease was observed in the levels of proteins (Fig. 2E, F) and genes (Fig. 2G) of enzymes related to milk fat synthesis. Similarly, synthesis of milk protein was also downregulated to varying degrees when AMPK was activated by AICAR and phenformin (Fig. 2H–K). In general, these results demonstrated that glucose starvation might inhibit milk fat and protein synthesis through AMPK signaling pathway.

### Energy stress inhibits milk fat and protein synthesis partially through AMPK

**Fig. 3:**
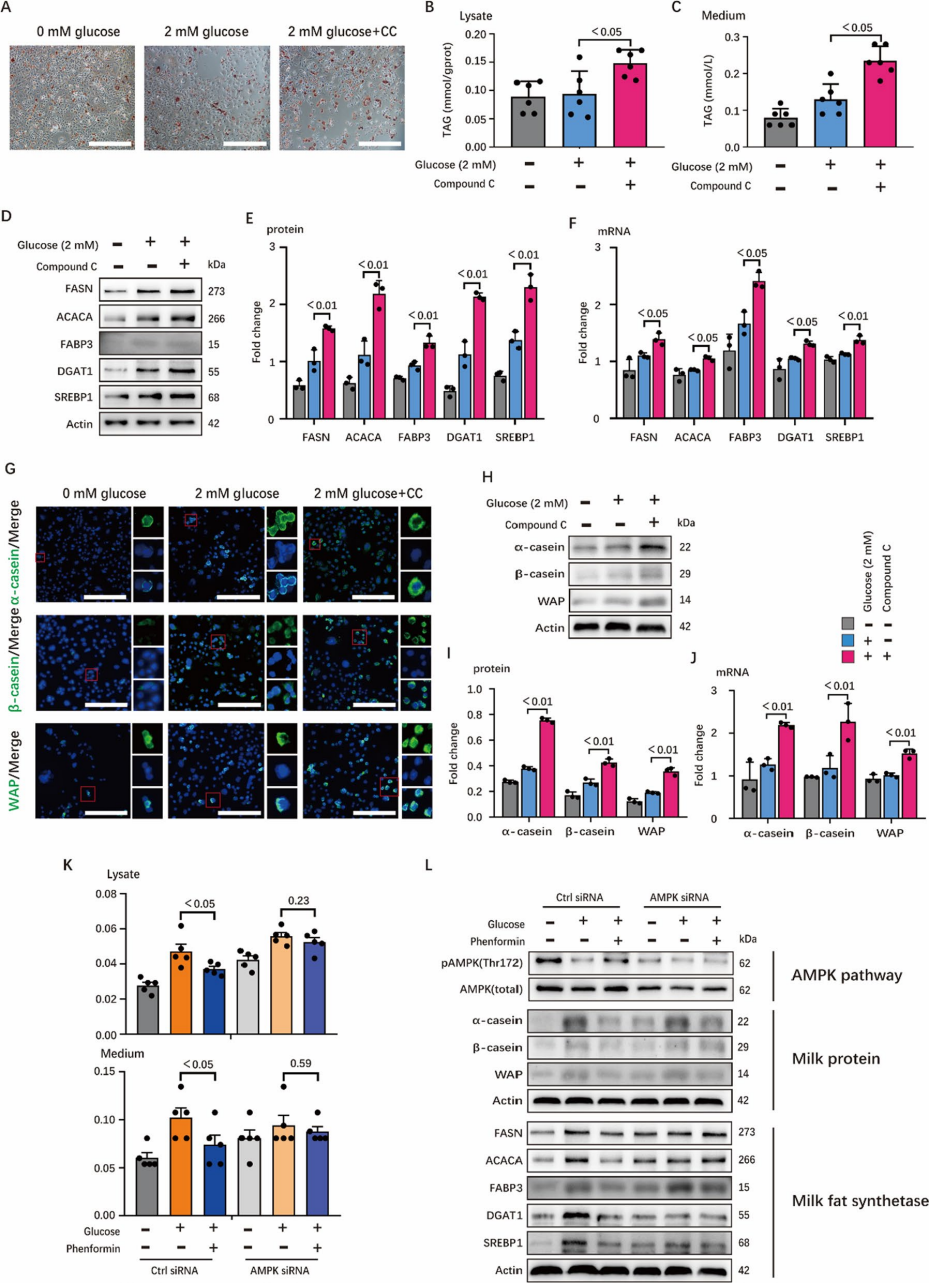
Inhibition of AMPK promotes the synthesis of milk fat and protein in HC11. **A** Oil red O staining images of HC11. Scale bars are 100 μm. **B** and **C** TAG concentration in cell (**B**) and medium (**C**), n = 6. **D**–**F** Western blots of HC11 lysates. In (**D**), bands were incubated with target proteins including FASN, ACACA, FABP3, DGAT1, SREBP1 and Actin antibodies, n = 3. Protein expressions of target proteins are shown in (**E**), n = 3. **F** Relative mRNA expressions of target proteins, n = 3. **G** Confocal microscopy of HC11 immunostained for α-casein (green), β-casein (green), WAP (green) and DAPI (blue). Scale bars, 100 μm. The small red boxes in the figure are typical areas selected, and the three small images on the right are the enlarged images of the target protein, nucleus and merge images in selected areas, respectively. **H**–**J** Western blots and real time PCR of HC11 lysates for critical protein synthesis genes. In (**H**), bands were incubated with target proteins including α-casein, β-casein, WAP and Actin antibodies, n = 3. Protein expressions of target proteins are shown in (**I**), n = 3. (**J)** shows relative mRNA expressions of target proteins, n = 3. **K** TAG concentration in cell (top) and medium (bottom), n = 6. Cells are first transfected with siRNA and treated with the corresponding medium, the cell lysate and medium are then collected for detection. **L** Western blots of HC11 lysates for AMPK signaling pathway, critical milk protein and fat synthesis genes. In this figure, cells for oil red O staining, TAG determination, immunofluorescence staining and WB were collected after 24 h incubation with 0 mM glucose medium, 2 mM glucose medium and (2 mM glucose + 50 μM Compound C) medium and cells for real-time PCR are 12 h. All datas with error bars are averages ± SEM. In histograms, the numbers above the column are the p-values marked as a range if significantly different (*P* < 0.05) and each small black dot represents a test value

To confirm the role of AMPK in energy stress-induced decrease of milk synthesis, we studied the role of AMPK inhibitor Compound C in milk synthesis under glucose starvation. As fatty acid synthesis might require glucose as substrates, 2 mM glucose was used to establish the slight glucose starvation model (Additional file 1: Fig. S2A), which could both activate AMPK and supply basic level of glucose as substrates. As expected, Compound C alleviated the negative effects of glucose starvation on milk fat synthesis (Fig. 3A–C) and enhanced the expression of proteins and genes of key enzymes (Fig. 3D–F). Immunofluorescent staining also showed that AMPK inhibition stimulated milk protein synthesis (Fig. 3G). Similar tendency was also observed in genes and proteins that related to milk protein synthesis (Fig. 3H–J). To further test whether the AMPK activity regulates milk fat and protein synthesis under energy stress, we knocked down AMPK using small interfering RNA (siRNA). The negative effects of AMPK on milk fat and protein synthesis were partially rescued when AMPK was knocked down (Fig. 3K, L). Intriguingly, milk fat and protein were significantly increased when HC11 was directly treated with compound c under severe energy deprivation (0 mM glucose) (Additional file 1: Fig. S2B–E), which indicates that other substrates might replace glucose for milk synthesis (as we will discuss below). Taken together, energy stress regulates milk fat and protein synthesis partially through AMPK signaling.

### AMPK signaling regulates milk synthesis partially through mTORC1 pathway

mTORC1 is a master regulator of cell growth and metabolism. Activation of mTORC1 further phosphorylates S6K1 and 4EBP1 and promotes protein synthesis, which might partially regulate milk fat and protein synthesis. In HC11 cell line, glucose starvation and/or AMPK agonists (AICAR and Phenformin) both inhibited mTORC1 signaling (phosphorylation of S6K1 and 4EBP1) (Additional file 1: Fig. S3A–B). Besides, glucose starvation also regulates the AMPK/mTORC1 pathway in PMEC (Additional file 1: Fig. S3C–D). In contrast, AMPK antagonist (Compound C) activated the mTORC1 signaling pathway (Additional file 1: Fig. S3E–F). To further investigate the role of mTORC1 on milk fat and protein synthesis, we pretreated the HC11 cell line with rapamycin (an inhibitor of mTORC1) to inhibit mTORC1 pathway (Additional file 1: Fig. S4A-B) before glucose stimulation. Likewise, the positive effects of glucose on the expression of critical enzymes involved in milk fat (Additional file 1: Fig. S4C–H) and protein synthesis (Additional file 1: Fig. S4I–K) was inhibited. Thus, this evidence indicates the critical role of mTORC1 in milk synthesis.

**Fig. 4:**
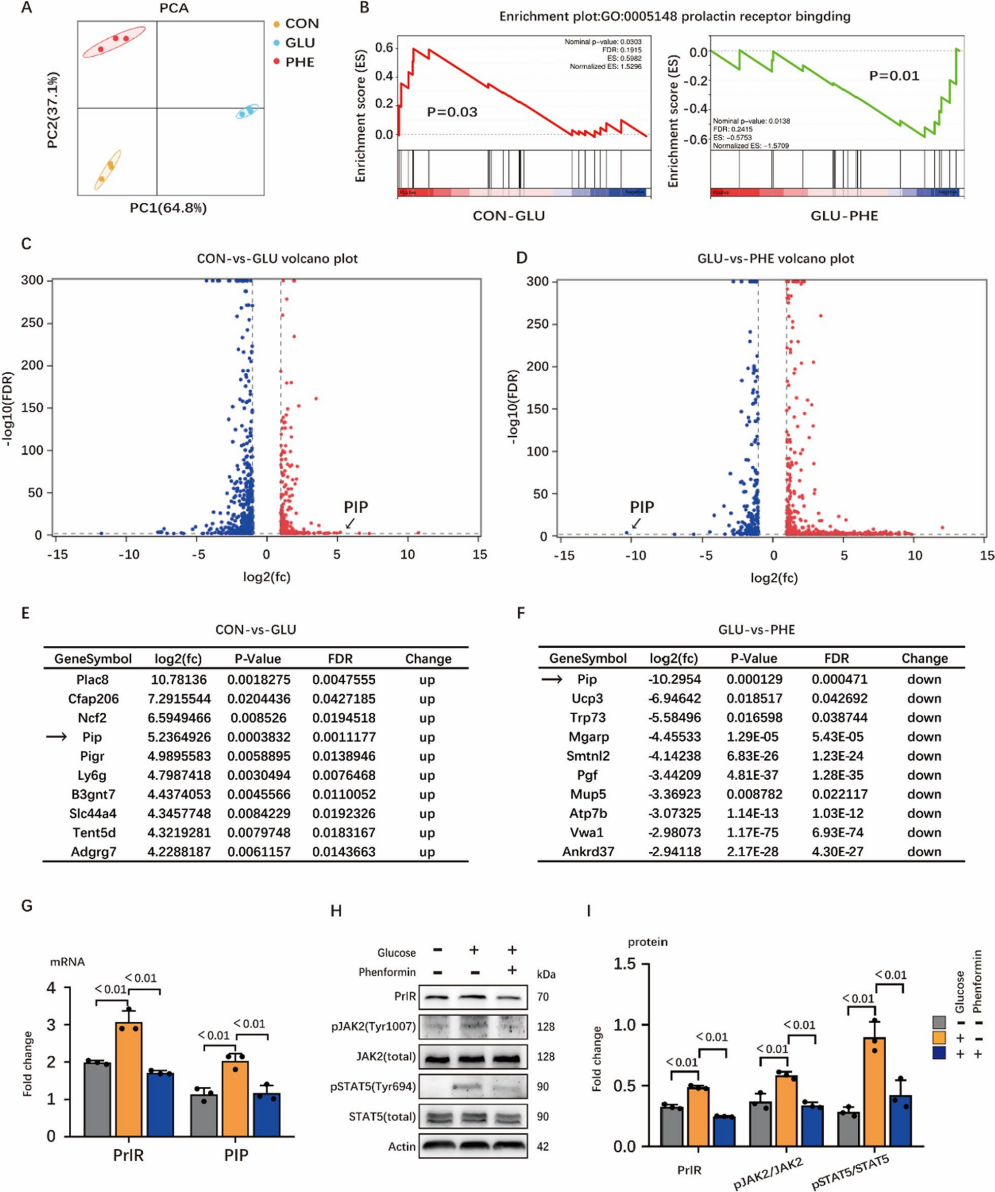
Activated AMPK induces the downstream PrlR/JAK2/STAT5/PIP pathway. **A** PCA of RNA-seq, n = 3. **B** GSEA showing enrichment of prolactin receptor binding in HC11 differentially expressed genes. The graph (left) shows the result of the control group-vs-glucose group and another graph (right) shows glucose group-vs-PHE group. **C** and **D** Volcano plots based on the significantly different genes in each comparison group. **C** Volcano plot of the control group-vs-glucose group and **D** Glucose group-vs-PHE group. The dots that the arrows point to are PIP. **E** and **F** (**E**) are top 10 significantly up-regulated 10 genes in the con-vs-glu comparison, and (**F**) are top 10 significantly down-regulated in the glu-vs-PHE comparison. **G** Relative mRNA expressions of PrlP and PIP, n = 3. **H** and **I** Western blots of HC11 lysates for PrlR/JAK2/STAT5 pathway. In (**H**), bands were incubated with target proteins and Actin antibodies, n = 3. Protein expressions of target proteins are shown in (**I**), n = 3. In this figure, cells for WB were collected after 24 h incubation with 0 mM glucose medium, 10 mM glucose medium and (10 mM glucose + 300 μM Phe) medium and cells for real-time PCR and RNA-seq are 12 h. All data with error bars are averages ± SEM. In histograms, the numbers above the column are the p-values marked as a range if significantly different (*P* < 0.05) and each small black dot represents a test value

### Energy stress might regulate milk synthesis through AMPK/PrlR pathway

In order to discover novel mechanism of how energy stress regulates milk synthesis, RNA-seq was performed to compare the transcriptomes of HC11 cell line starved for glucose or treated with AMPK agonist with those under normal situation (Fig. 4A). GSEA analysis indicated that signaling pathways related to prolactin binding were significantly inhibited in group CON (glucose starvation) and group PHE (AMPK activation) (Fig. 4B). Intriguingly, the fold change of prolactin induced protein (PIP, considered as a downstream of PrlR signaling pathway) was prominent in the volcano map (Fig. 4C, D) and was ranked fourth and first in terms of up- and down-regulated genes in Fig. 4C, D, respectively (Fig. 4E, F). Therefore, we made a hypothesis that AMPK activation might decrease PrlR expression and inhibit its downstream signaling. Real-time PCR confirmed the decrease of PrlR and PIP in group CON and group PHE (Fig. 4G). Furthermore, the phosphorylation of JAK2 and STAT5 (downstream targets of PrlR signaling) were also significantly inhibited under glucose starvation or AMPK activation (Fig. 4H, I). We concluded that energy stress may regulate milk synthesis through PrlR pathway due to the significant change on AMPK/PrlR and its downstream pathways under AMPK activation.

Intriguingly, RNA-seq and GSEA analysis found that genes (4F2hc, xCT, CAT1, ASCT1, ASCT2) involved in amino acid transportation were up-regulated under glucose starvation and stimulation of phenformin (Additional file 1: Fig. S5A–B). These RNA-seq data were then confirmed by RT-PCR (Additional file 1: Fig. S5C). Even though amino acids were largely transported into cells, milk protein synthesis was still inhibited under glucose starvation and stimulation of phenformin, which was shown by our previous results. These evidences indicated that amino acids might not be used for milk synthesis, but stead metabolized for energy supply. Amino acid transaminase catalyzes the transfer of the amino group (-NH2), which converts amino acids into *α*-keto acid for further metabolism. RNA-seq data and RT-PCR data further confirmed that multiple amino acid transaminases (BCAT2, GPT2, GOT1) were up-regulated with under glucose starvation (Additional file 1: Fig. S5D–E), which indicates that these amino acids could be used as energy supply.

**Fig. 5:**
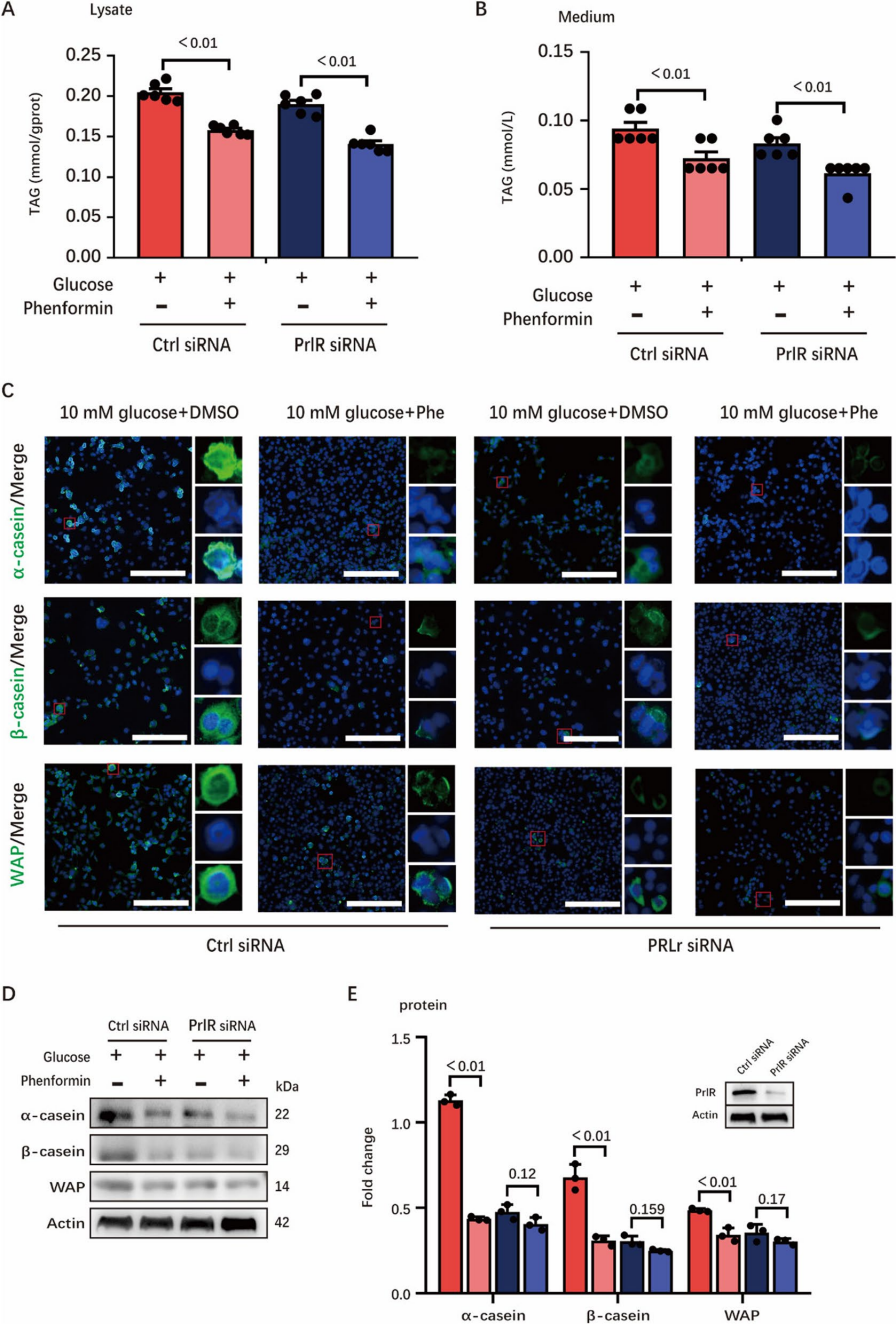
Knock-down of PrlR lowers synthesis of milk protein but little affects milk fat. **A** and **B** TAG concentration in cell (**A**) and medium (**B**), n = 6. **C** Confocal microscopy of HC11 immunostained for α-casein (green), β-casein (green), WAP (green) and DAPI (blue). Scale bars, 100 μm. The small red boxes in the figure are typical areas selected, and the three small images on the right are the enlarged images of the target protein, nucleus and merge images in selected areas, respectively. **D** and **E** Western blots of HC11 lysates for critical milk protein synthesis genes. **D** Representative western-blotting bands hybridized with target proteins including α-casein, β-casein, WAP and Actin antibodies, n = 3. **E** Result of analyzing the WB bands. In this figure, cells (transfected by siRNA in advance) for TAG determination, immunofluorescence staining and WB were collected after 24 h incubation with 10 mM glucose medium and (10 mM glucose + 300 μM Phe) medium. All datas with error bars are averages ± SEM. In histograms, the numbers above the column are the p-values marked as a range if significantly different (*P* < 0.05) and each small black dot represents a test value

### AMPK activation plays a role in the ubiquitination-induced degradation of PrlR by up-regulating the activity of β-TrCP

In order to confirm whether the PrlR signaling is involved in AMPK-regulated milk fat and protein synthesis, we knocked down PrlR using siRNA. After PrlR knockdown, AMPK activation still inhibited milk fat synthesis (Fig. 5A, B). Nevertheless, the synthesis of milk protein was significantly suppressed after PrlR knockdown as is shown by immunofluorescence images (Fig. 5C). Besides, we also observed similar trends at the protein level (Fig. 5D, E). Thus, PrlR seems to promote the synthesis of milk protein but not milk fat.

**Fig. 6:**
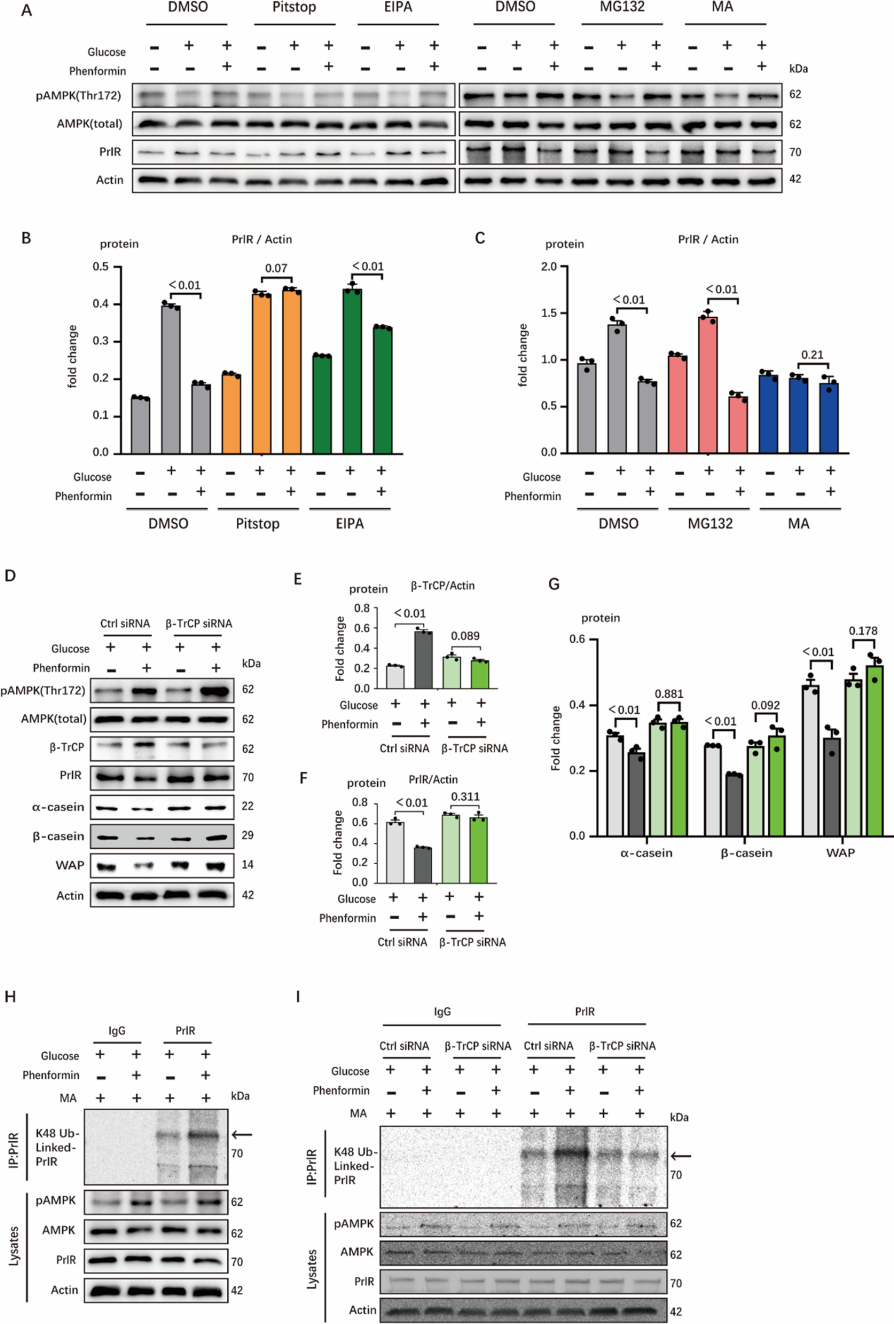
β-TrCP mediating AMPK is involved in the ubiquitination degradation of PrlR. **A**–**C** Western blots of HC11 lysates for PrlR, n = 3. As shown in the left part of (**A**), the cells were treated with DMSO, Pitstop and EIPA to explore the effect of AMPK activation on the degradation of PrlR to explore the degradation mode of PrlR. In the right part of (**A**), similar methods have also been used to explore the organelles involved in the degradation of PrlR. (**B)** and (**C)** are the quantifications of protein expression in the above two experiments, respectively. (Pitstop: pinocytosis inhibitor, EIPA: endocytosis inhibitor, MG132: proteasome inhibitor, MA: lysosomal inhibitor) **D**–**G** Western blots of HC11 lysates for AMPK/β-TrCP/PrlR pathway, n = 3. In (**D**), bands were incubated with indicated proteins and Actin antibodies, n = 3. Protein expressions of β-TrCP, PrlR and milk proteins are shown in (**E**), (**F**) and (**G**), respectively, n = 3. **H** Immunoblot analysis of PrlR immunoprecipitates in HC11, probed for AMPK/Ub-PrlR pathway. After being cultured in the designated medium (all containing MA), cells were lysed by CHAPS lysis buffer, and lysate were not only used for immunoprecipitation to detect the ubiquitination of PrlR but also for WB to detect AMPK/PrlR pathway. **I** Immunoblot analysis of PrlR immunoprecipitates in HC11, probed AMPK/β-TrCP/Ub-PrlR pathway. After being transinfected by siRNA and cultured in the designated medium (all containing MA), cells were lysed by CHAPS lysis buffer, and lysate were not only used for immunoprecipitation to detect the ubiquitination of PrlR but also for WB to detect AMPK/PrlR pathway. In this figure, cells for WB were collected after 24 h incubation with indicated medium. All datas with error bars are averages ± SEM. In histograms, the numbers above the column are the *p*-values marked as a range if significantly different (*P* < 0.05) and each small black dot represents a test value

PrlR is a transmembrane protein, its degradation could be regulated by a variety of physiological processes (such as ubiquitination) and multiple organelles (such as proteasomes and lysosomes). Thus, we then studied the degradation mechanism of PrlR. The results showed that PrlR seems to be taken up more through endocytosis and finally be degraded by lysosomes (Fig. 6A–C). F-box protein β-transducin repeat-containing protein (β-TrCP) is a component of the ubiquitin ligase complex that triggers ubiquitin-dependent degradation of multiple proteins. We found that β-TrCP was significantly up-regulated under AMPK activation, which is accompanied with the degradation of PrlR (Fig. 6D, E). Importantly, when the β-TrCP was knocked down by siRNA, its induction of PrlR degradation was reduced (Fig. 6D–F). Consistent with this, the expression level of milk protein also decreased with the knockdown of β-TrCP (Fig. 6D–G). In order to confirm that the degradation of PrlR may be due to β-TrCP-induced ubiquitination, we used MA (lysosomal inhibitor) to block the degradation process, and used immunoprecipitation to observe the ubiquitination of PrlR. We found that AMPK activation can significantly promote the ubiquitination of PrlR (Fig. 6H). In subsequent experiments, we found that knockdown of β-TrCP can weaken the ubiquitination of PrlR (Fig. 6I). In summary, AMPK activation can promote the degradation of PrlR under lysosome endocytosis, specifically by up-regulating the activity of β-TrCP to regulate ubiquitination-induced degradation of PrlR.

### AMPK activation up-regulates the expression of PGC-1α and enhances the β-oxidation of fatty acids by inhibiting PGC-1α acetylation

**Fig. 7:**
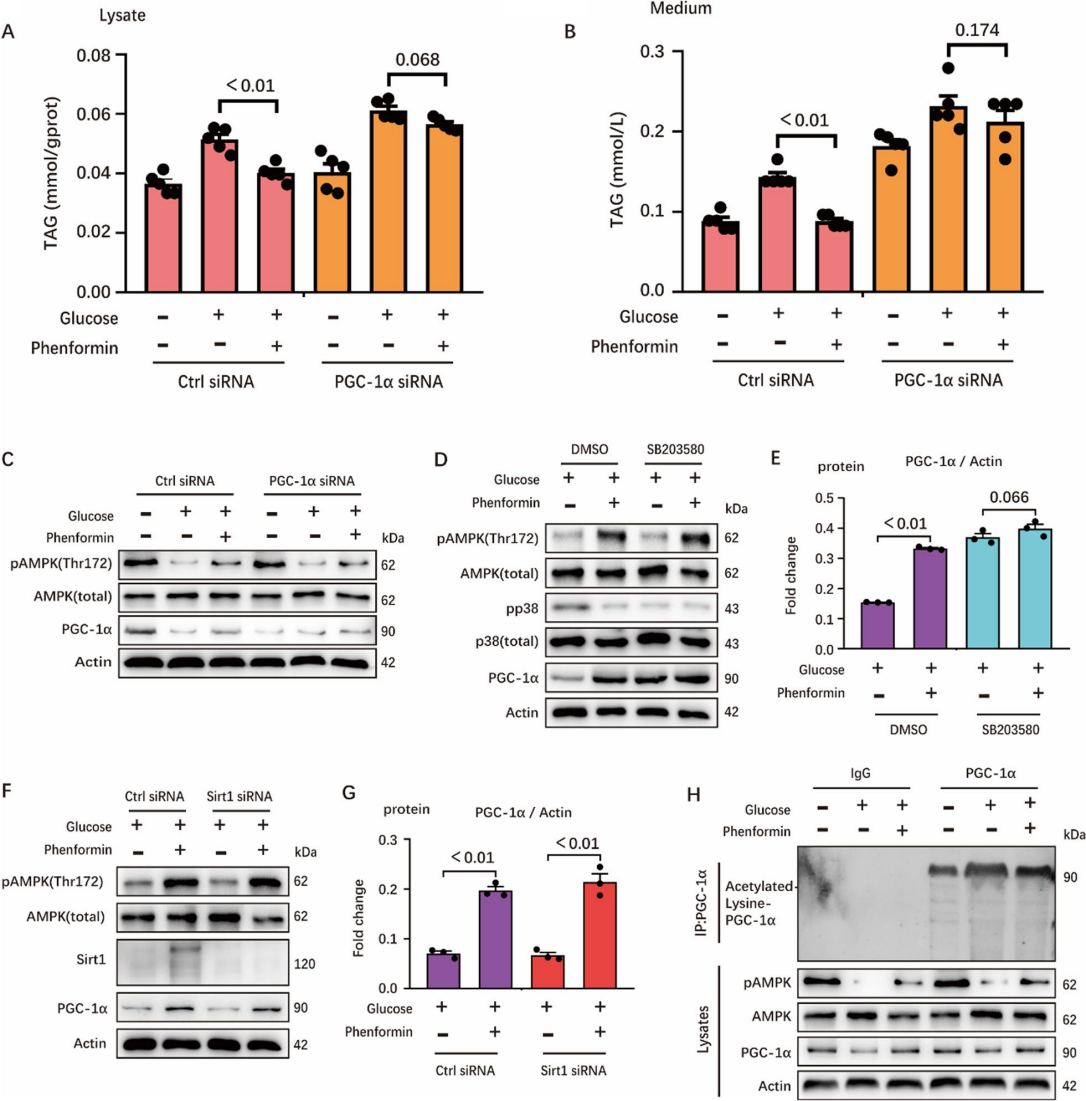
Loss of PGC-1α facilitates milk fat production due to its acetylation regulated by AMPK. **A** and **B** TAG concentration in cell (**A**) and medium (**B**), n = 5. In the experiment, cells (transfected by siRNA in advance) for TAG determination were collected after 24 h incubation with 0 mM glucose medium, 10 mM glucose medium and (10 mM glucose + 300 μM Phe) medium. **C** Western blots of HC11 lysates for AMPK/PGC-1α signaling pathway, n = 3. **D** and **E** Western blots of HC11 lysates for AMPK/p38/PGC-1α signaling pathway, n = 3. Cells were treated with 10 mM glucose medium and (10 mM glucose + 300 μM Phe) medium in the case of DMSO and SB203580 (p38 inhibitor) for 24 h, respectively. In (**D**), bands were incubated with target proteins and Actin antibodies, n = 3. Protein expressions of target proteins are shown in (**E**), n = 3. **F** and **G** Western blots of HC11 lysates for AMPK/Sirt1/PGC-1α signaling pathway, n = 3. Cells were treated with 10 mM glucose medium and (10 mM glucose + 300 μM Phe) medium in the case of control-knockdown and Sirt1-knockdown for 24 h, respectively. In (**F**), bands were incubated with target proteins and Actin antibodies, n = 3. Protein expressions of target proteins are shown in (**G**), n = 3. **H** Immunoblot analysis of PGC-1α immunoprecipitates in HC11, probed for acetylation of PGC-1α. Cells were lysed by CHAPS lysis buffer, and lysate were not only used for immunoprecipitation to detect the acetylation of PGC-1α but also for WB to detect AMPK/PGC-1α pathway. In this figure, cells for TAG determination and WB were collected after 24 h incubation with indicated medium. All datas with error bars are averages ± SEM. In histograms, the numbers above the column are the *p*-values marked as a range if significantly different (*P* < 0.05) and each small black dot represents a test value

In order to identified how AMPK regulates milk fat level, we considered to study the process of fatty acids synthesis and fatty acid β-oxidation. Previously, ACC (critical enzyme involved in fatty acid synthesis) and PGC-1α (critical enzyme involved in fatty acid β-oxidation) were identified as downstream targets of AMPK and found to play significant roles in fat metabolism in hepatocyte [12] and adipocyte [13]. Since a number of evidences have already indicated that ACC can affect fat production in mammary gland. Here, we would illustrate the impact of AMPK on milk fat synthesis from the perspective of fatty acid oxidation, thus, we took PGC-1α as the entry point. Subsequent tests further proved that the negative effect of AMPK on milk fat synthesis was partially rescued when PGC-1α was knocked down (Fig. 7A–C). These evidence clearly indicated that AMPK might regulate milk fat synthesis mainly through PGC-1α in mammary epithelial cells.

Since previous studies have found that AMPK regulates PGC-1α through p38 MAPK [14] or Sirt1 [15] in cancer cells and skeletal muscle, we further investigated the relationship between AMPK and PGC-1α in HC11. We found that AMPK activation inhibits the phosphorylation of p38 pathway. When p38 was inhibited, the expression of PGC-1α was increased independent of the activation of AMPK (Fig. 7D, E). Regarding Sirt1, although AMPK activation promotes Sirt1 expression, Sirt1 knockdown has no effect on PGC expression induced by AMPK (Fig. 7F, G). In order to explore its mechanism, we used immunoprecipitation to observe the relationship between AMPK and the acetylation of PGC-1α. We found that glucose starvation or AMPK activation decreased the acetylation level of PGC-1α (Fig. 7H). Collectively, AMPK might regulate milk fat synthesis partially by up-regulating the activity of PGC-1α and inhibiting its acetylation.

## Discussion

The intimate relationship between milk synthesis and AMPK signaling has been identified for a long time [16], whereas the underlying mechanism is not well characterized. Here, we identified two novel target proteins (PrlR and PGC-1α) of AMPK signaling that are involved in the regulation of milk synthesis. Mechanistically, AMPK triggers the ubiquitination and degradation of PrlR through β-TrCP, which then attenuates the PrlR-JAK2-STAT5 pathway involved in milk protein synthesis. At the same time, AMPK decreases the acetylation process of PGC-1α and inhibits milk fat accumulation.

PrlR signaling regulates the growth and maturation of the mammary gland, which is also critical for the maintenance of lactation [17]. PrlR deficiency significantly inhibits milk protein synthesis. In this study, RNA-seq data revealed that the gene expression of PrlR was down-regulated when AMPK was activated, which indicates AMPK might regulate milk protein synthesis through PrlR. Ubiquitination is one of the crucial mechanism to induce the degradation of hormone receptors [18]. Studies have shown that AMPK could control the uptake of long-chain fatty acids (LCFA) by regulating the expression of the membrane receptor CD36 through Parkin-mediated polyubiquitination in intestinal epithelial cells [19]. Furthermore, the activation of AMPK triggers the interaction between β-TrCP and Glioma-associated oncogenes (GLI1), which induces β-TrCP-mediated GLI1-ubiquitination and degradation in human brain cancer cells [20]. Our results first revealed that PrlR was mainly degraded by lysosome through endocytosis process under the influence of AMPK activation. In the above process, AMPK activation up-regulated the activity of β-TrCP, which induced the ubiquitination degradation of PrlR.

PGC-1α is considered as a transcriptional coactivator of PPARγ [21], which regulates fatty acid β-oxidation in the liver [22] and skeletal muscle [23]. To date, the role of PGC-1α in mammary gland is still unclear. In this study, we found AMPK regulates milk fat production not only through the process of milk fat synthesis, but also the process of β-oxidation. Previously, AMPK activation has been reported to activate CaMKKβ and increase the expression of PGC-1α [24]. In addition, more evidences also indicate AMPK might regulate the expression of PGC-1α through Sirt1 [25] or p38 MAPK dependent signaling [14]. Surprisingly, in this study, we observed that AMPK regulates activity of PGC-1α mainly through the p38 MAPK signaling pathway, but not Sirt1 signaling in mammary epithelial cells. The inconsistent results regarding the regulatory molecules that signal AMPK to PGC-1α could partially be explained by tissue-specific regulation system. To our knowledge, the activity of PGC-1α is regulated by post-translational modifications, including phosphorylation [26] and acetylation [27]. In subsequent experiments, we provided evidence that AMPK can inhibit the acetylation of PGC-1α. AMPK activation inhibits the activity of ACACA, which makes it difficult for acetyl-CoA to be converted into fatty acids. Thus, acetyl-CoA accumulation might eventually lead to the inhibition of the acetylation reaction [28]. As a non-histone protein, the molecular mechanism of acetylation of PGC-1α warrants additional work.

## Conclusions

**Fig. 8:**
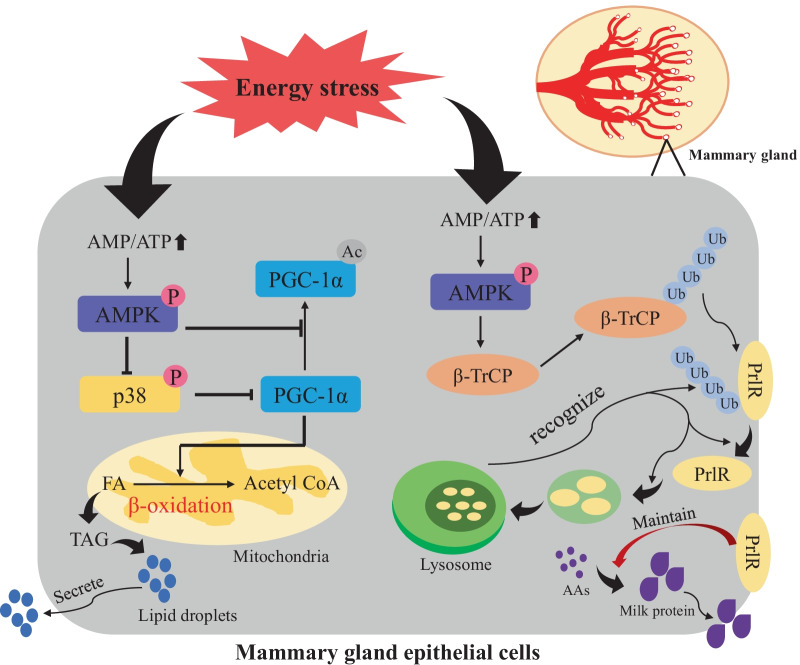
Overall of AMPK’s effect on milk fat and protein in HC11 cell line

In summary, we confirmed the hypothesis that AMPK regulates the production of milk protein and fat in the mammary gland by regulating the degradation of PrlR and the acetylation of PGC-1α, respectively (Fig. 8). Although some mechanisms require further verification, it is foreseeable that this mechanism can be utilized to provide new possibilities and inspiration for the regulation of milk synthesis.

## Supplementary Information

**Additional file 1**. **Fig. S1.** Glucose increases milk fat production but inhibits AMPK. (A) Oil red O staining images of pMECs. Scale bars are 100 μm. (B and C) Western blots of pMECs lysates for critical fat synthesis genes. (B) are representative western-blotting bands hybridized with target proteins including FASN, ACACA, FABP3, DGAT1, SREBP1 and Actin antibodies, n=3. (C) is the result of analyzing the WB bands, n=3. (D) Ratio of ATP and ADP, n=3. HC11 cells were collected after 24 h incubation with 0 mM and 10 mM glucose mediums. (E and F) Western blots analysis of HC11 lysates for AMPK signaling pathway, n=3. Cells were collected after 24 h incubation with 0 mM and 10 mM glucose mediums. **Fig. S2.** Inhibition of AMPK still promotes the synthesis of milk fat and protein in the absence of glucose. (A) Western blots analysis of HC11 lysates for AMPK signaling pathway. Cells were collected after 24 h incubation with 0 mM glucose medium, 10 mM glucose medium, (10 mM glucose +CC) medium, 2 mM glucose medium and (2 mM glucose +CC) medium. (B and C) TAG TAG concentration in cell (B) and medium (C), n=6. (D and E) Western blots of HC11 lysates for critical protein synthesis genes. (D) are representative western-blotting bands hybridized with target proteins including α-casein, β-casein, WAP and Actin antibodies, n=3. (E) is the result of analyzing the WB bands, n=3. **Fig. S3.** AMPK activates the mTORC1/S6K1/4EBP1 pathway in both HC11 and pMECs. (A and B) Western blots analysis of HC11 lysates for mTORC1/S6K1/4EBP1 signaling pathway, n=3. Cells were collected after 24 h incubation with 0 mM glucose medium, 10 mM glucose medium, (10 mM glucose +AICAR) medium and (10 mM glucose +Phe) medium. (C and D) Western blots analysis of pMECs lysates for AMPK/mTORC1 signaling pathway, n=3. (E and F) Western blots analysis of HC11 lysates for AMPK/mTORC1 signaling pathway. Cells were collected after 24 h incubation with 0 mM glucose medium, 2 mM glucose medium and (2 mM glucose +CC) medium, n=3. **Fig. S4.** mTORC1 up-regulates synthesis of milk fat and protein in HC11. (A and B) Western blots analysis of HC11 lysates for mTORC1/S6K1/4EBP1 signaling pathway, n=3. (C) Oil red O staining images of HC11. Scale bars are 50 μm. (D and E) TAG concentration in cell (D) and medium (E), n=4. (F-H) Western blots and real time PCR of HC11 lysates for critical fat synthesis genes. (F) are representative western-blotting bands hybridized with target proteins including FASN, ACACA, FABP3, DGAT1, SREBP1 and Actin antibodies, n=3. (G) is the result of analyzing the WB bands and (H) represents datas from real-time PCR, n=3. (I-K) Western blots and real time PCR of HC11 lysates for critical protein synthesis genes. (I) are representative western-blotting bands hybridized with target proteins including α-casein, β-casein, WAP and Actin antibodies, n=3. (J) is the result of analyzing the WB bands and (K) represents datas from real-time PCR, n=3. In this figure, cells for oil red O staining, TAG determination and WB were collected after 24 h incubation with 0 mM glucose medium, 10 mM glucose medium and (10 mM glucose +rapamycin) medium and cells for real-time PCR are 12 h. All datas with error bars are averages ± SEM. In histograms, the numbers above the column are the p-values marked as a range if significantly different (P < 0.05) and each small white dot represents a test value. **Fig. S5.** AMPK is involved in regulating the activity of amino acid transporters in HC11. (A) GSEA showing enrichment of amino acid transport across plasma membrane in HC11 differentially expressed genes. The graph (left) shows the result of the control group-vs-glucose group and another graph (right) shows glucose group-vs-PHE group. (B and C) (B) is heatmap analysis of up-regulated (red) or down-regulated genes (blue) in HC11, n=3. The tested genes are amino acid transporters selected from the GSEA gene set (shown in the figure).(C) is the result of real-time PCR, n=3. (D and E) (D) is heatmap analysis of up-regulated (red) or down-regulated genes (blue) in HC11, n=3. The tested genes are typical aminotransferases (shown in the figure). (E) is the result of real-time PCR, n=3. In this figure, cells for RNA-seq and real-time PCR were collected after 12 h incubation with 0 mM glucose medium, 10 mM glucose medium and (10 mM glucose +Phe) medium. All datas with error bars are averages ± SEM. In histograms, the numbers above the column are the p-values marked as a range if significantly different (P < 0.05) and each small white dot represents a test value.

## Data Availability

The data analyzed during the current study are available from the corresponding author on request.

## Abbreviations

AMPK: AMP-activated protein kinase
mTORC1: Mammalian target of rapamycin complex 1
PrlR: Prolactin receptor
β-TrCP: β-Transducin repeat-containing protein
PGC-1α: Peroxisome proliferator-activated receptor gamma coactivator-1 alpha
FBP: Fructose-1,6-bisphosphate
FA: Fatty acid
TAG: Triacylglycerol
ACACA: Acetyl-coA carboxylase alpha
SREBP1c: Sterol regulatory element-binding protein 1c
S6K1: S6 kinase 1
4EBP1: 4E binding protein 1
pMECs: Porcine mammary gland epithelial cells
TSC2: Tuberin 2
