# Supplementary material for: Energy deprivation-induced AMPK activation inhibits milk synthesis by targeting PrlR and PGC-1α
Source: Cell Commun Signal. 2022 Mar 5;20:25. doi: 10.1186/s12964-022-00830-6 (PMC8898430; doi:10.1186/s12964-022-00830-6)
Supplement: Supplementary file 2 — Additional file 1. Fig. S1. Glucose increases milk fat production but inhibits AMPK. (A) Oil red O staining images of pMECs. Scale bars are 100 μm. (B and C) Western blots of pMECs lysates for critical fat synthesis genes. (B) are representative western-blotting bands hybridized with target proteins including FASN, ACACA, FABP3, DGAT1, SREBP1 and Actin antibodies, n=3. (C) is the result of analyzing the WB bands, n=3. (D) Ratio of ATP and ADP, n=3. HC11 cells were collected after 24 h incubation with 0 mM and 10 mM glucose mediums. (E and F) Western blots analysis of HC11 lysates for AMPK signaling pathway, n=3. Cells were collected after 24 h incubation with 0 mM and 10 mM glucose mediums. Fig. S2. Inhibition of AMPK still promotes the synthesis of milk fat and protein in the absence of glucose. (A) Western blots analysis of HC11 lysates for AMPK signaling pathway. Cells were collected after 24 h incubation with 0 mM glucose medium, 10 mM glucose medium, (10 mM glucose +CC) medium, 2 mM glucose medium and (2 mM glucose +CC) medium. (B and C) TAG TAG concentration in cell (B) and medium (C), n=6. (D and E) Western blots of HC11 lysates for critical protein synthesis genes. (D) are representative western-blotting bands hybridized with target proteins including α-casein, β-casein, WAP and Actin antibodies, n=3. (E) is the result of analyzing the WB bands, n=3. Fig. S3. AMPK activates the mTORC1/S6K1/4EBP1 pathway in both HC11 and pMECs. (A and B) Western blots analysis of HC11 lysates for mTORC1/S6K1/4EBP1 signaling pathway, n=3. Cells were collected after 24 h incubation with 0 mM glucose medium, 10 mM glucose medium, (10 mM glucose +AICAR) medium and (10 mM glucose +Phe) medium. (C and D) Western blots analysis of pMECs lysates for AMPK/mTORC1 signaling pathway, n=3. (E and F) Western blots analysis of HC11 lysates for AMPK/mTORC1 signaling pathway. Cells were collected after 24 h incubation with 0 mM glucose medium, 2 mM glucose medium and (2 mM glucose +CC) medium [file 12964_2022_830_MOESM2_ESM.pdf]

Supplementary Information

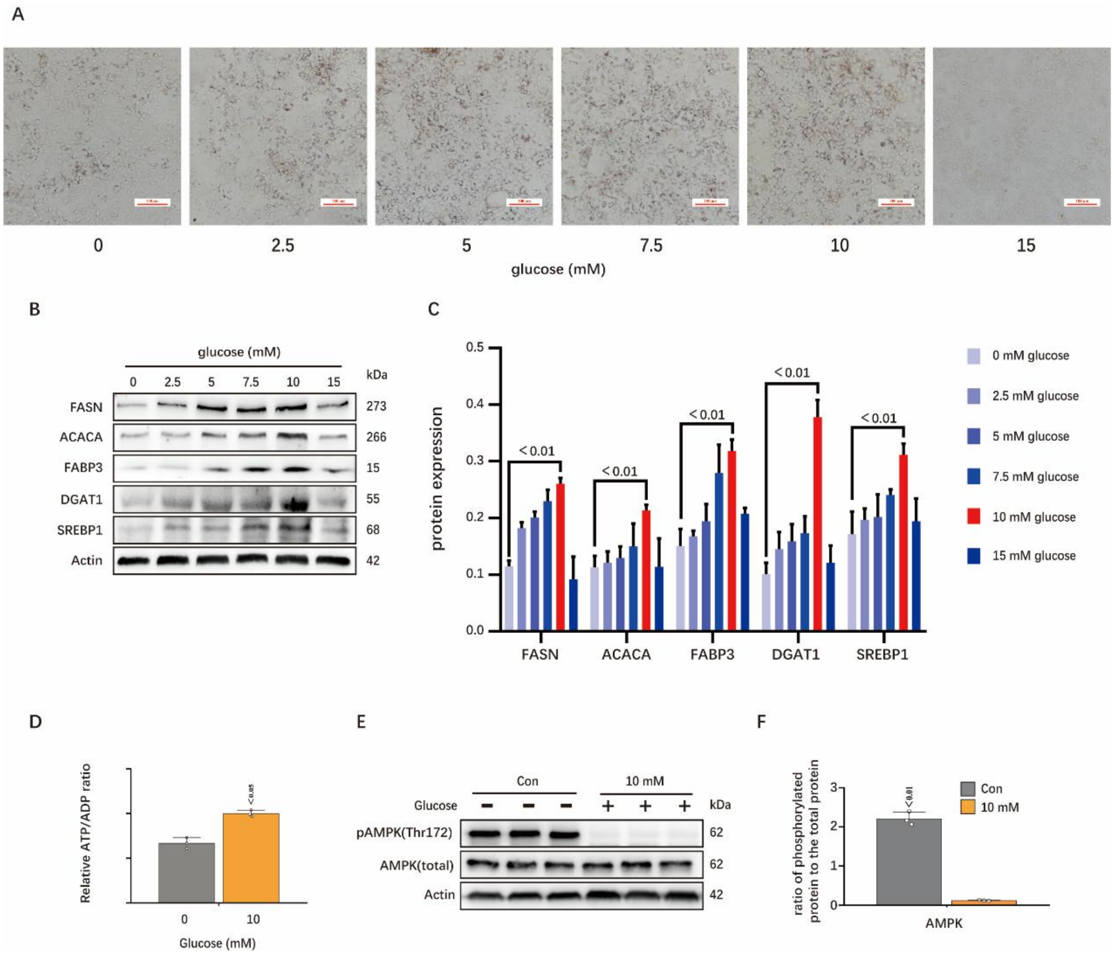

Fig. S1 Glucose increases milk fat production but inhibits AMPK. (A) Oil red O staining images of pMECs. Scale bars are 100 μm. (B and C) Western blots of pMECs lysates for critical fat synthesis genes. (B) are representative western-blotting bands hybridized with target proteins including FASN, ACACA, FABP3, DGAT1, SREBP1 and Actin antibodies, n=3. (C) is the result of analyzing the WB bands, n=3. (D) Ratio of ATP and ADP, n=3. HC11 cells were collected after 24 h incubation with 0 mM and 10 mM glucose mediums. (E and F) Western blots analysis of HC11 lysates for AMPK signaling pathway, n=3. Cells were collected after 24 h incubation with 0 mM and 10 mM glucose mediums.

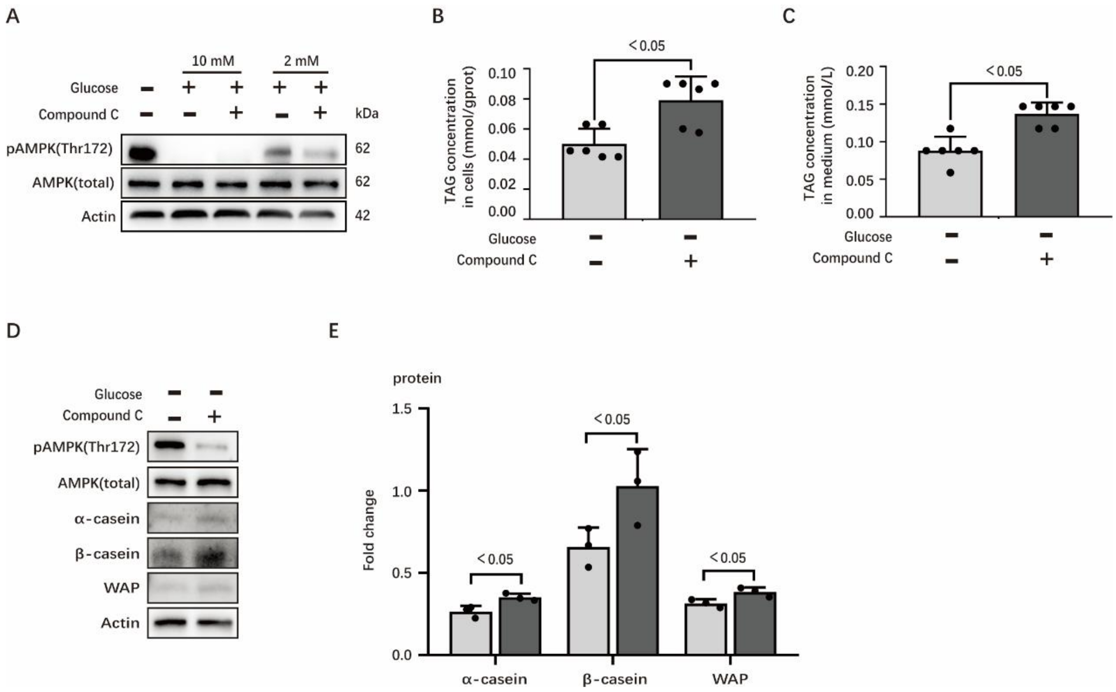

Fig. S2 Inhibition of AMPK still promotes the synthesis of milk fat and protein in the absence of glucose. (A) Western blots analysis of HC11 lysates for AMPK signaling pathway. Cells were collected after 24 h incubation with 0 mM glucose medium, 10 mM glucose medium, (10 mM glucose +CC) medium, 2 mM glucose medium and (2 mM glucose +CC) medium. (B and C) TAG TAG concentration in cell (B) and medium (C), n=6. (D and E) Western blots of HC11 lysates for critical protein synthesis genes. (D) are representative western-blotting bands hybridized with target proteins including α-casein, β-casein, WAP and Actin antibodies, n=3. (E) is the result of analyzing the WB bands, n=3.

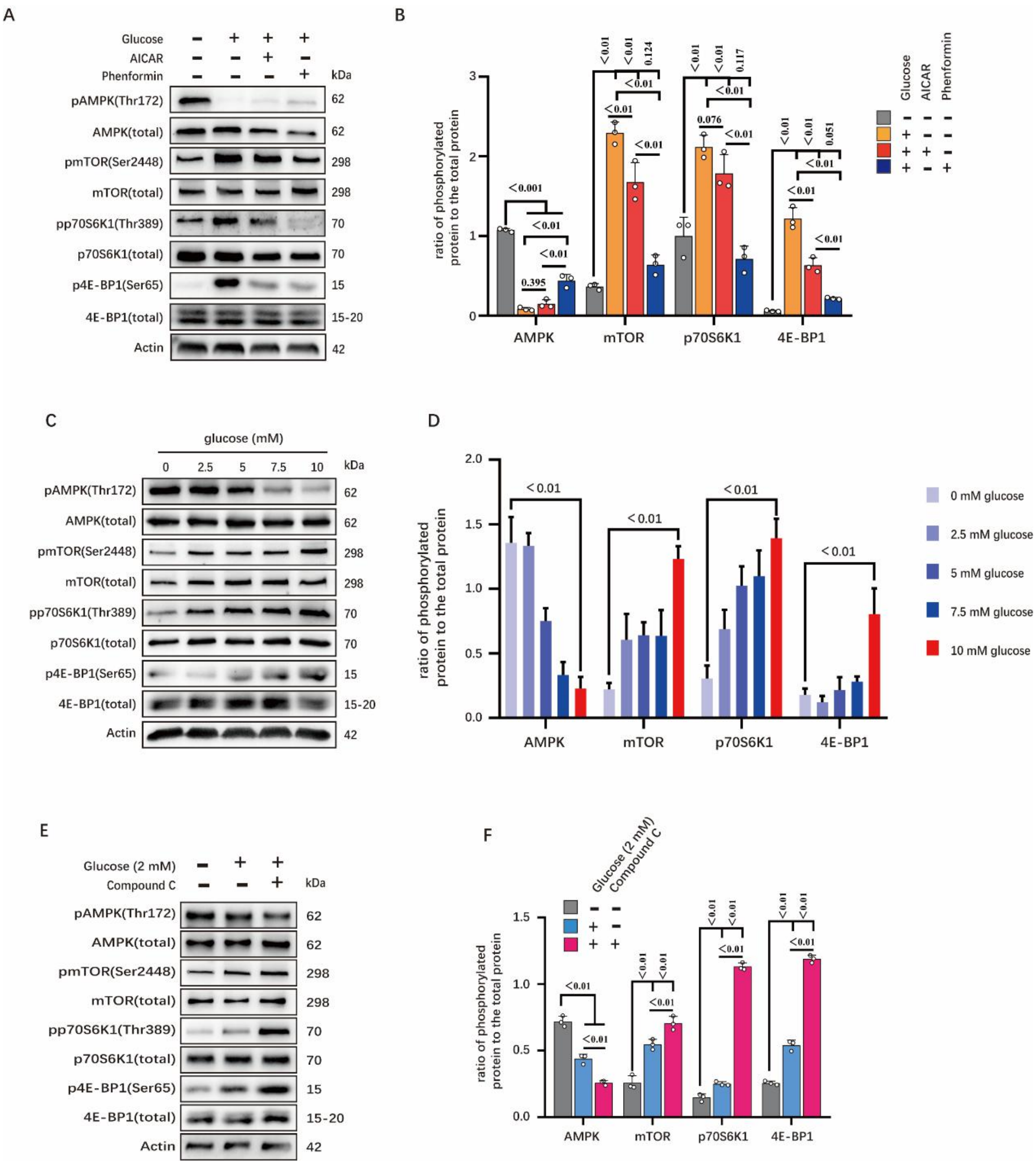

Fig. S3 AMPK activates the mTORC1/S6K1/4EBP1 pathway in both HC11 and pMECs. (A and B) Western blots analysis of HC11 lysates for mTORC1/S6K1/4EBP1 signaling pathway, n=3. Cells were collected after 24 h incubation with 0 mM glucose medium, 10 mM glucose medium, (10 mM glucose +AICAR) medium and (10 mM glucose +Phe) medium. (C and D) Western blots analysis of pMECs lysates for AMPK/mTORC1 signaling pathway, n=3. (E and F) Western blots analysis of HC11 lysates for AMPK/mTORC1 signaling pathway. Cells were collected after 24 h incubation with 0 mM glucose medium, 2 mM glucose medium and (2 mM glucose +CC) medium, n=3.

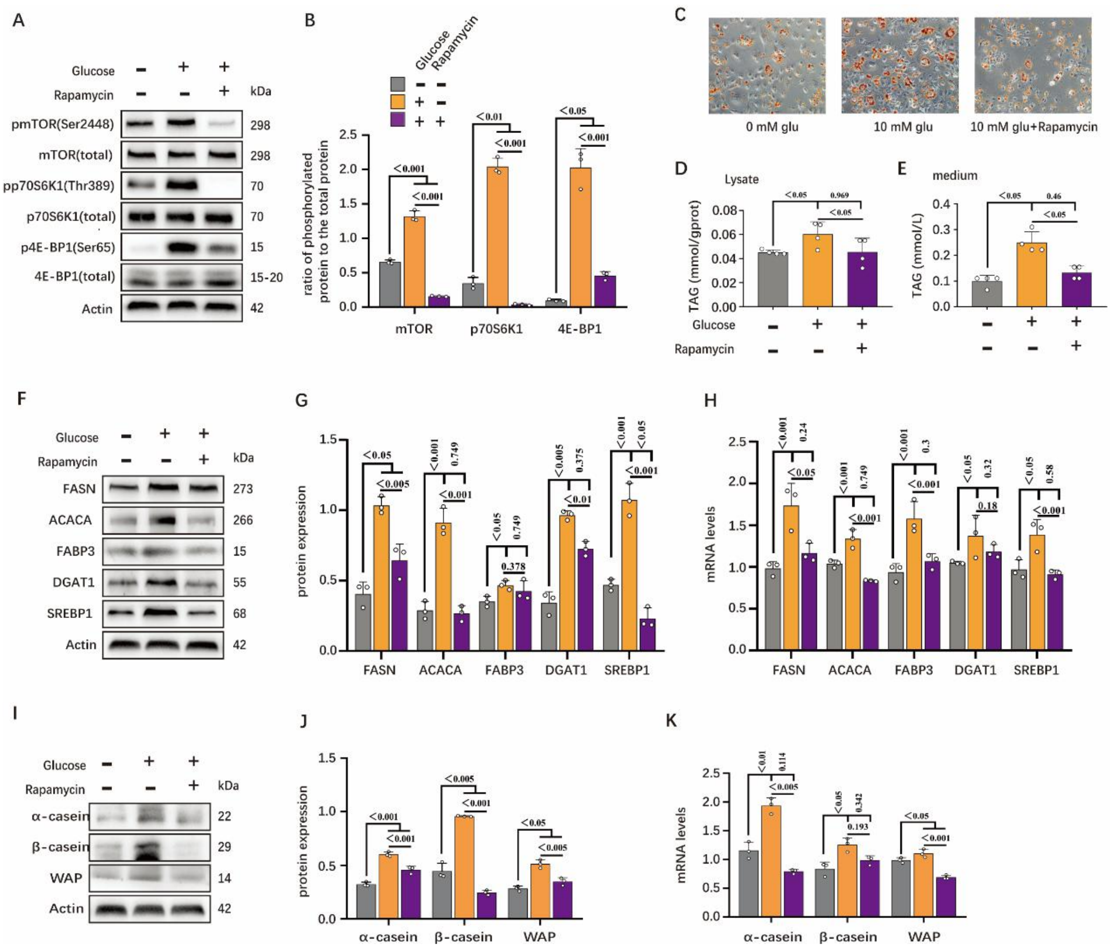

Fig. S4 mTORC1 up-regulates synthesis of milk fat and protein in HC11. (A and B) Western blots analysis of HC11 lysates for mTORC1/S6K1/4EBP1 signaling pathway, n=3. (C) Oil red O staining images of HC11. Scale bars are 50 μm. (D and E) TAG concentration in cell (D) and medium (E), n=4. (F-H) Western blots and real time PCR of HC11 lysates for critical fat synthesis genes. (F) are representative western-blotting bands hybridized with target proteins including FASN, ACACA, FABP3, DGAT1, SREBP1 and Actin antibodies, n=3. (G) is the result of analyzing the WB bands and (H) represents datas from real-time PCR, n=3. (I-K) Western blots and real time PCR of HC11 lysates for critical protein synthesis genes. (I) are representative western-blotting bands hybridized with target proteins including α-casein, β-casein, WAP and Actin antibodies, n=3. (J) is the result of analyzing the WB bands and (K) represents datas from real-time PCR, n=3. In this figure, cells for

oil red O staining, TAG determination and WB were collected after 24 h incubation with 0 mM glucose medium, 10 mM glucose medium and (10 mM glucose +rapamycin) medium and cells for real-time PCR are 12 h. All datas with error bars are averages ± SEM. In histograms, the numbers above the column are the p-values marked as a range if significantly different ($P < 0.05$) and each small white dot represents a test value.

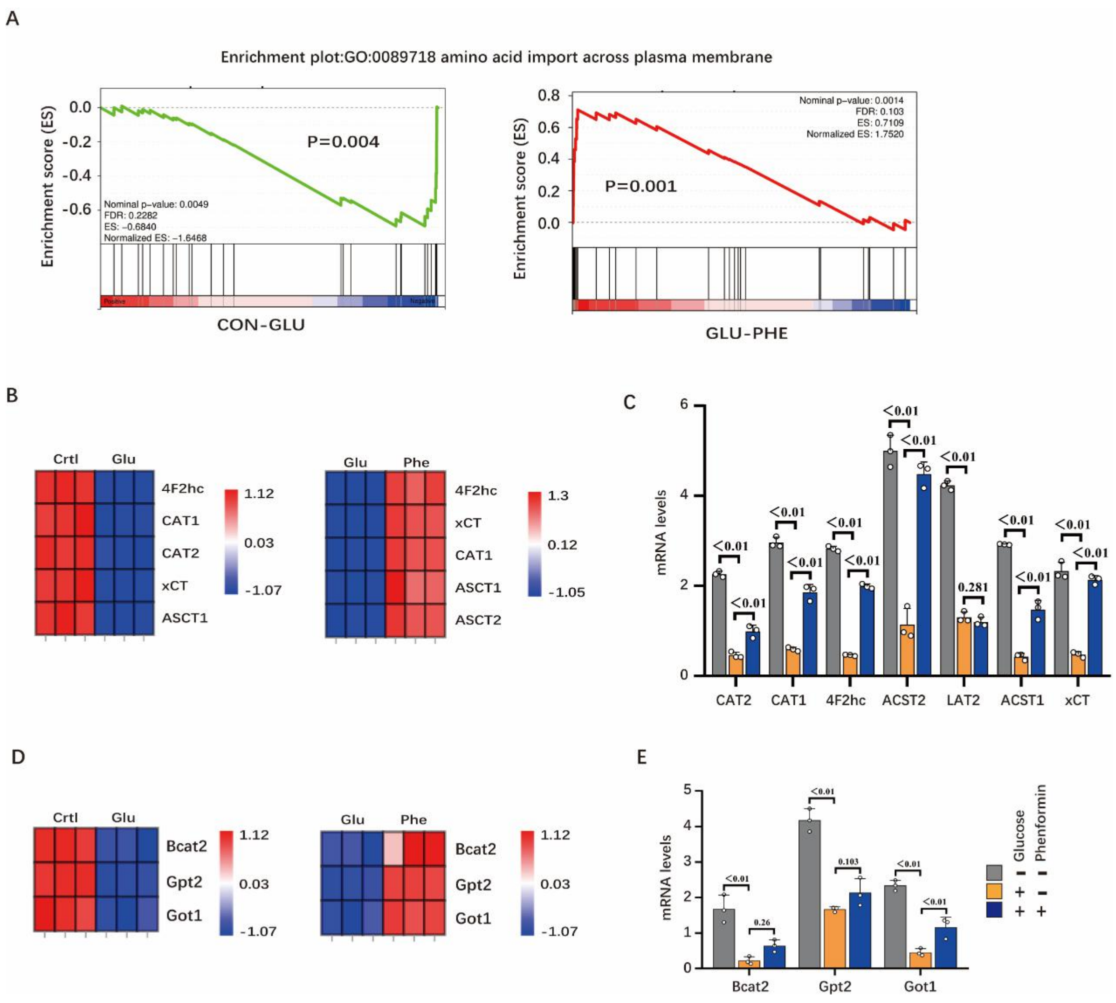

Fig. S5 AMPK is involved in regulating the activity of amino acid transporters in HC11. (A) GSEA showing enrichment of amino acid transport across plasma membrane in HC11 differentially expressed genes. The graph (left) shows the result of the control group-vs-glucose group and another graph (right) shows glucose group-vs-PHE group. (B and C) (B) is heatmap analysis of up-regulated (red) or down-regulated genes (blue) in HC11, n=3. The tested genes are amino acid transporters selected from the GSEA gene set (shown in the figure). (C) is the result of real-time PCR, n=3. (D and E) (D) is heatmap analysis of up-regulated (red) or down-regulated genes (blue) in HC11, n=3. The tested genes are typical aminotransferases (shown in the figure). (E) is the result of real-time PCR, n=3. In this figure, cells for RNA-seq and real-time PCR were collected after 12 h incubation with 0 mM glucose medium, 10 mM glucose medium and (10 mM glucose +Phe) medium. All datas with error bars are averages ± SEM. In

histograms, the numbers above the column are the p-values marked as a range if significantly different (P＜0.05) and each small white dot represents a test value.
